# Supplementary material for: Myonectin protects against skeletal muscle dysfunction in male mice through activation of AMPK/PGC1α pathway
Source: Nat Commun. 2023 Aug 4;14:4675. doi: 10.1038/s41467-023-40435-2 (PMC10403505; doi:10.1038/s41467-023-40435-2)
Supplement: Supplementary file 1 — Supplementary Information [file 41467_2023_40435_MOESM1_ESM.pdf]

Supplementary Information for

Myonectin protects against skeletal muscle dysfunction in male mice through activation of AMPK/PGC1α pathway

Yuta Ozaki [1], Koji Ohashi* [2], Naoya Otaka [1], Hiroshi Kawanishi [1], Tomonobu Takikawa [1], Lixin Fang [1], Kunihiko Takahara [1], Minako Tatsumi [2], Sohta Ishihama [1], Mikito Takefuji [1], Katsuhiro Kato [1], Yuuki Shimizu [1], Yasuko K Bando [1], Aiko Inoue [3], Masafumi Kuzuya [3,4], Shinji Miura [5], Toyoaki Murohara [1] and Noriyuki Ouchi* [2]

[1] Department of Cardiology, Nagoya University Graduate School of Medicine, Nagoya, Japan

[2] Department of Molecular Medicine and Cardiology, Nagoya University Graduate School of Medicine, Nagoya, Japan

[3] Institute of Innovation for Future Society, Nagoya University Graduate School of Medicine, Nagoya, Japan

[4] Department of Community Healthcare & Geriatrics, Nagoya University Graduate School of Medicine, Nagoya, Japan

[5] Laboratory of Nutritional Biochemistry, Graduate School of Nutritional and Environmental Sciences, University of Shizuoka, Shizuoka, Japan

*Corresponding author.

Email: ohashik@med.nagoya-u.ac.jp or nouchi@med.nagoya-u.ac.jp

This PDF file includes:

Supplementary Figures 1 to 17

Supplementary Table 1

Supplementary Figure 1

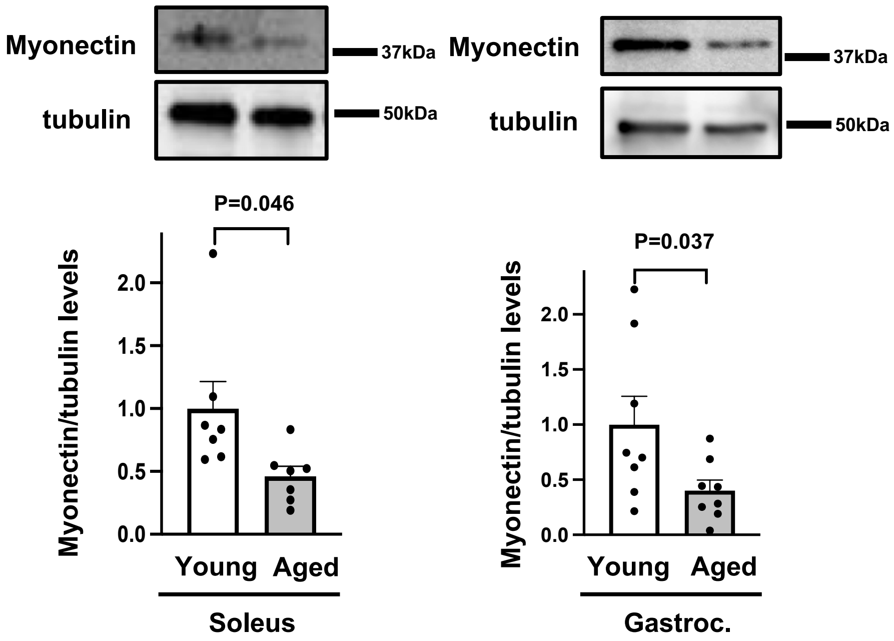

Supplementary Figure 1. Aged mice have reduced protein levels of myonectin in skeletal muscle. The protein levels of myonectin and tubulin were evaluated by Western blot analysis. Upper panels show representative blots of myonectin and tubulin in soleus and gastrocnemius (Gastroc.) muscles of 20-week-old young and 80-week-old aged WT mice. Lower panels show quantitative analyses of myonectin/tubulin signal ratios in soleus and gastrocnemius muscles of young and aged WT mice. N=7 in each group. Data are presented as means ± SEM. Two-tailed unpaired Student's t-test was performed.

Supplementary Figure 2

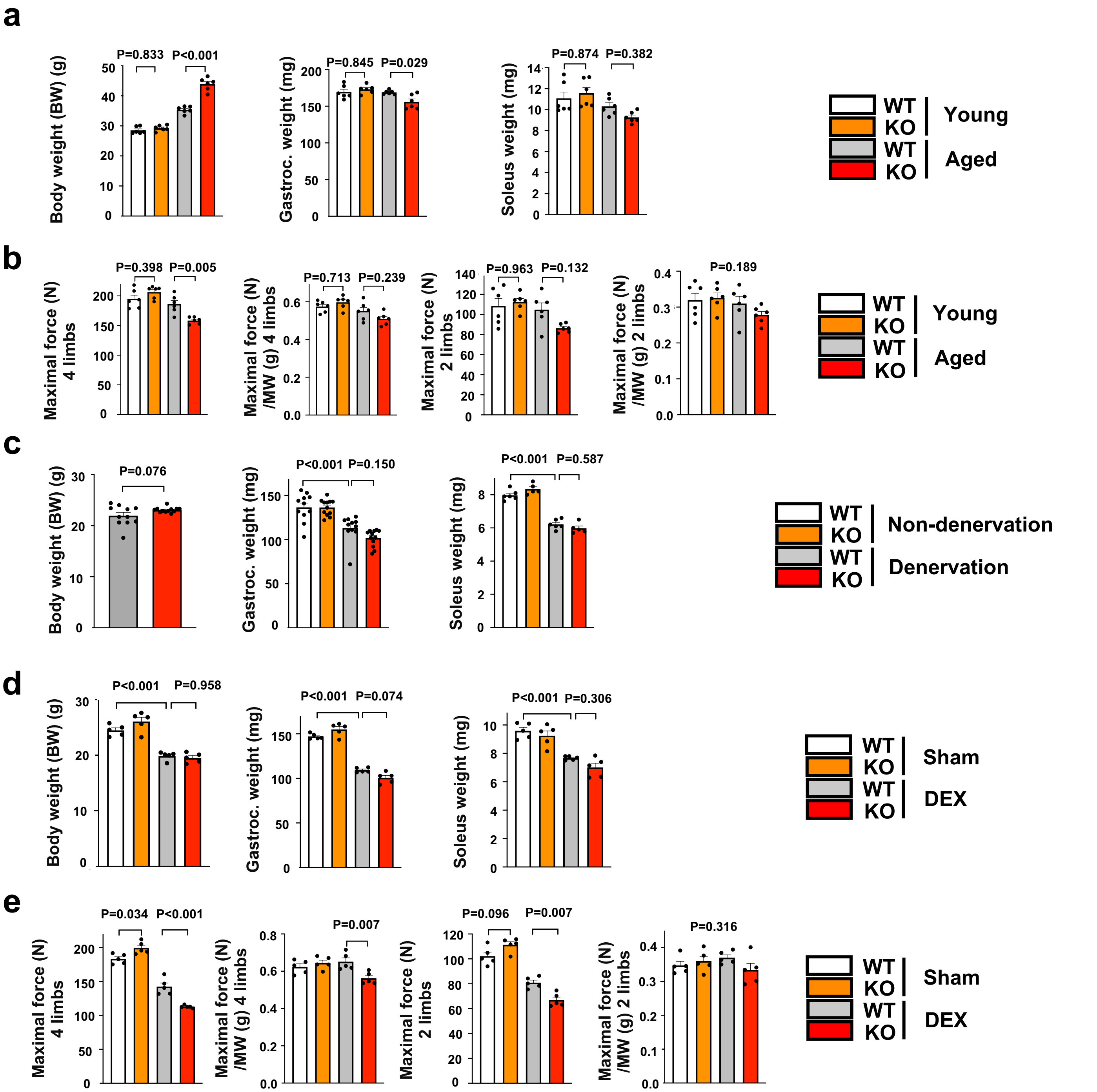

Supplementary Figure 2. Characteristics of myonectin-KO and WT mice in various models of muscle atrophy. a, Body weight and skeletal muscle (Gastrocnemius (Gastroc.) and Soleus) weight of 20-week-old young WT mice, 20-week-old young myonectin-KO mice, 80-week-old aged WT mice and 80-week-old aged myonectin-KO mice. N=6 in each group. **b,** Maximal grip strength in 4 limbs or fore 2 limbs which is non-normalized or normalized by gastrocnemius muscle weight (MW) of 20-week-old young WT mice, 20-week-old young myonectin-KO mice, 80-week-old aged WT mice and 80-week-old aged myonectin-KO mice. N=6 in each group. **c,** Left panel shows body weight of WT and myonectin-KO mice after denervation. Middle panel shows non-denervated or denervated gastroc. muscle weight of WT and myonectin-KO mice. WT: N=11, myonectin-KO: N=12. Right panel shows non-denervated or denervated soleus muscle weight of WT and myonectin-KO mice. N=6 in each group. **d,** Body weight and skeletal muscle (Gastroc. and Soleus) weight of WT and myonectin-KO mice after treatment with vehicle (Sham) or dexamethasone (DEX). N=5 in each group. **e**, Maximal grip strength in 4 limbs or fore 2 limbs which is non-normalized or normalized by gastrocnemius muscle weight (MW) of WT and myonectin-KO mice after treatment with sham or DEX is shown. N=5 in each group. Data are presented as means ± SEM. Two-tailed unpaired Student's t-test (**c, left panel**) and one-way ANOVA with a post-hoc analysis **(a,b,c, middle and right panels, d,e)** were performed.

Supplementary Figure 3

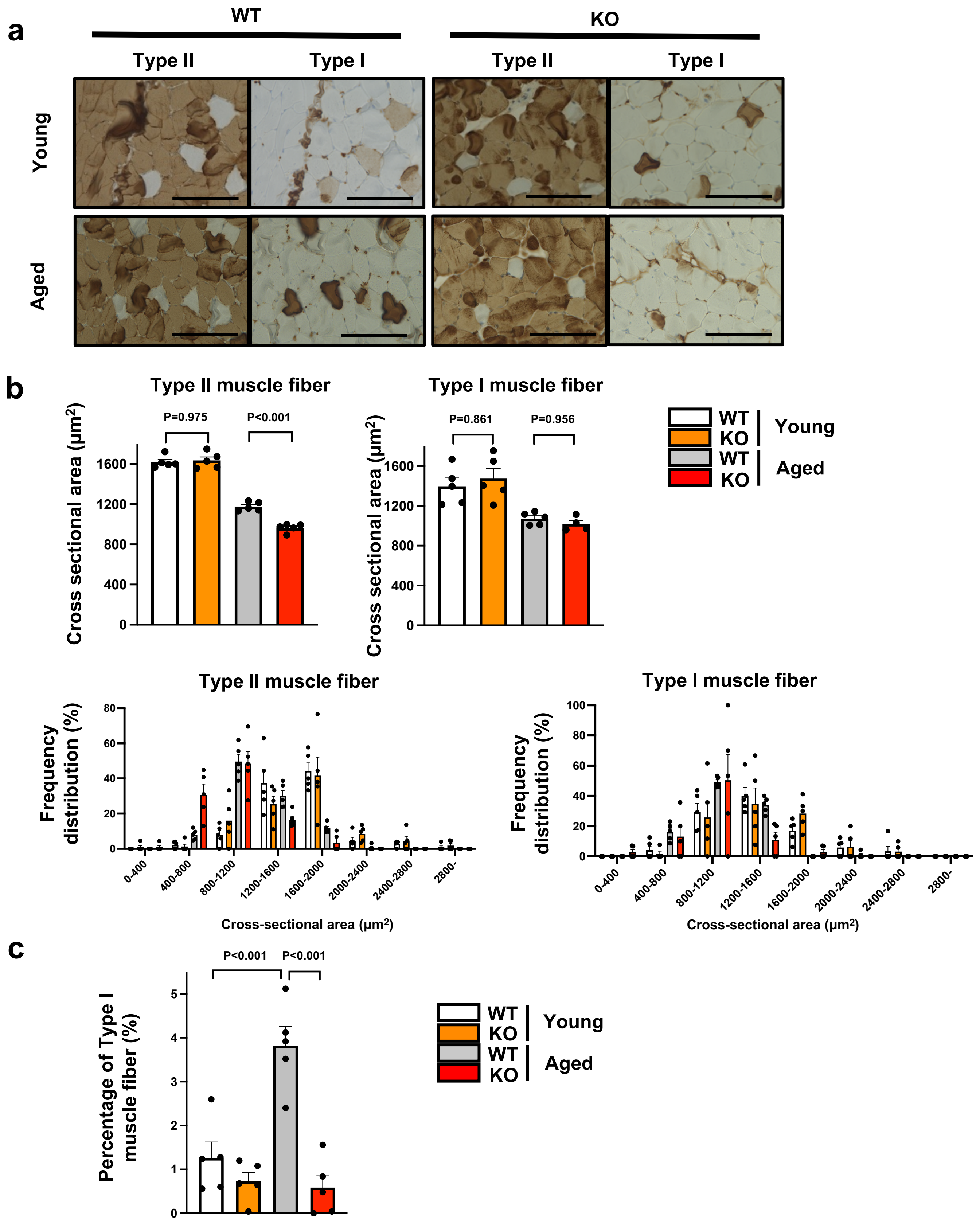

Supplementary Figure 3. Myonectin deficiency promotes atrophy of Type II muscle fiber in aged mice. Muscle fibers of Type I or Type II in gastrocnemius muscles of 20-week-old young WT mice, 20-week-old young myonectin-KO mice, 80-week-old aged WT mice and 80-week-old aged myonectin-KO mice, were evaluated by immune-histochemical staining. **a,** Representative immunostaining photos of Type II and Type I fibers. Scale bars show 100μm. **b,** Quantitative analysis of mean cross sectional area (CSA) and CSA distribution of Type II and Type I fibers. N=5 in each group. **c,** The ratio of Type I fiber number to total muscle fiber. N=5 in each group. Data are presented as means ± SEM. One-way ANOVA with a post-hoc analysis **(b, upper panels, c)** was performed.

Supplementary Figure 4

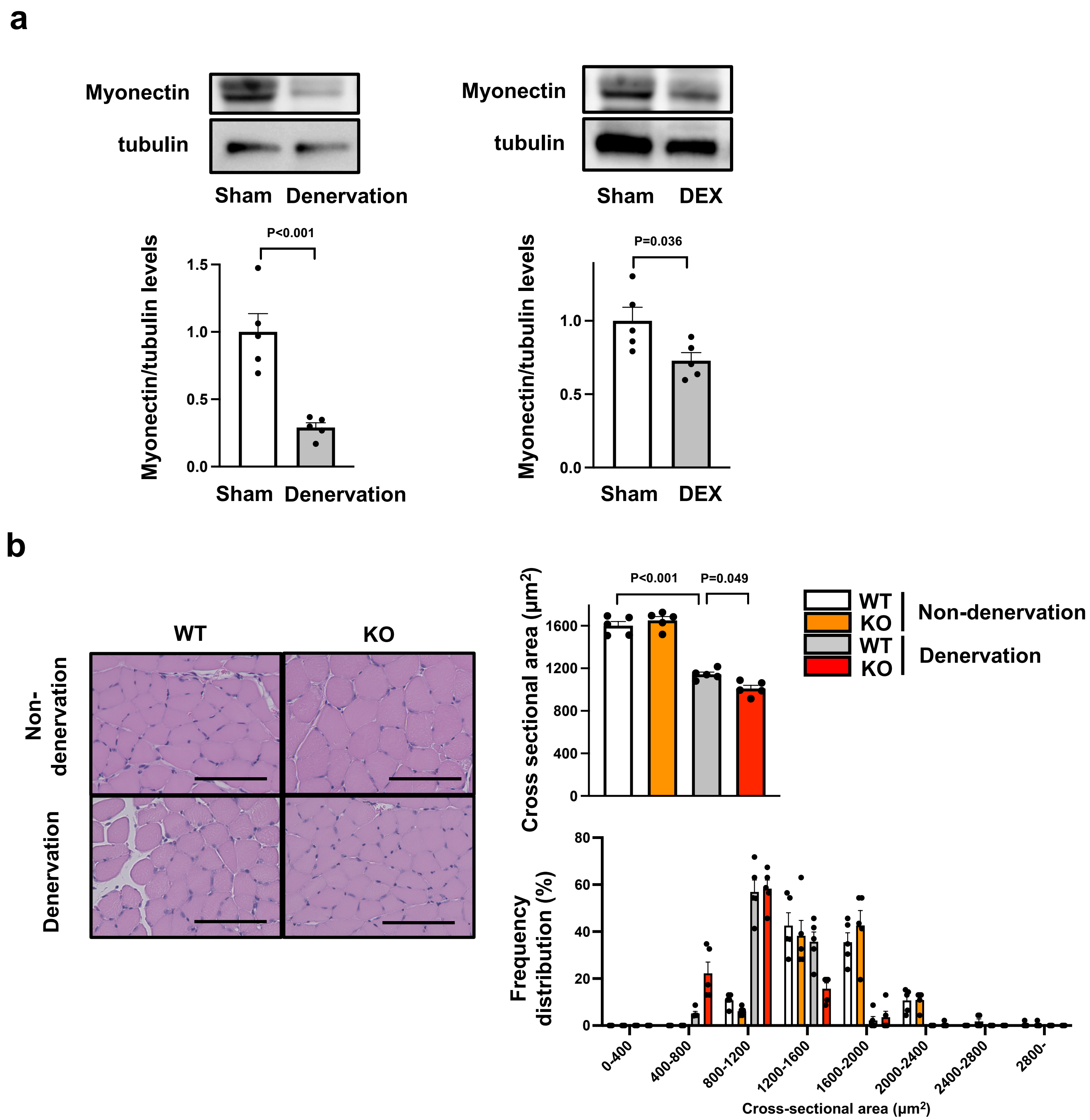

Supplementary Figure 4. Reduced expression of myonectin in skeletal muscle of WT mice by denervation or dexamethasone treatment. a, The protein levels of myonectin and tubulin were evaluated by Western blot analysis. Upper panels show representative blots of myonectin and tubulin in gastrocnemius muscles after sham treatment, denervation or dexamethasone (DEX) treatment. Lower panels show quantitative analysis of myonectin/tubulin signal ratios in gastrocnemius muscles after sham, denervation operation or DEX treatment. N=5 in each group. **b,** Left panels show representative cross sectional images of soleus muscle. Scale bars show 100 μm. Right panels show quantitative analysis of mean cross sectional area (CSA) and CSA distribution. N=5 in each group. Data are presented as means ± SEM. Two-tailed unpaired Student's t-test (**a**) and one-way ANOVA with a post-hoc analysis **(b, upper right panel)** were performed.

Supplementary Figure 5

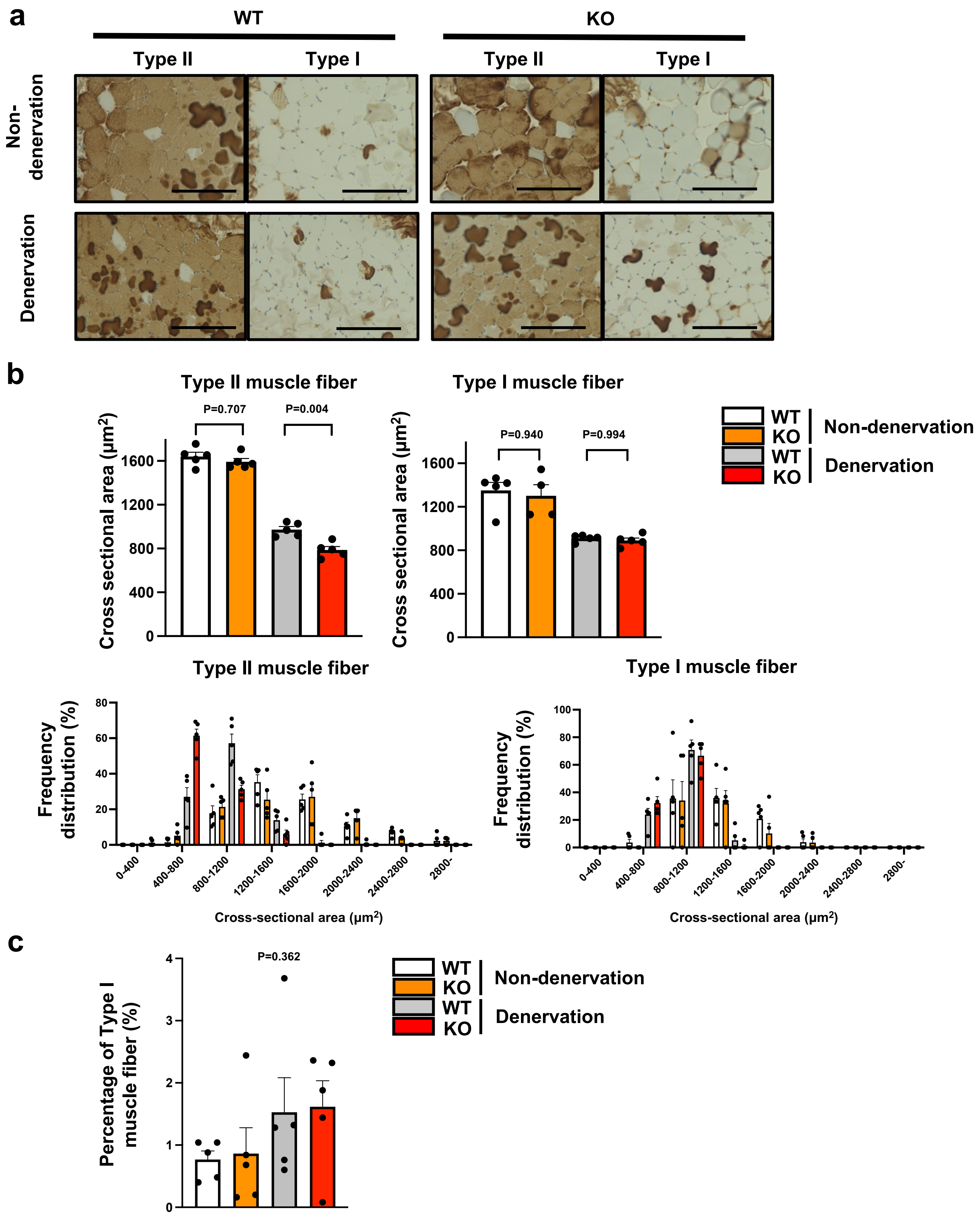

Supplementary Figure 5. Myonectin deficiency promotes denervation-induced atrophy of Type II muscle fiber in gastrocnemius muscle. Muscle fibers of Type I or Type II in denervated or non-denervated gastrocnemius muscles of WT and myonectin KO mice, were evaluated by immune-histochemical staining. **a,** Representative immunostaining photos of Type II and Type I fibers. Scale bars show 100μm. **b,** Quantitative analysis of mean cross sectional area (CSA) and CSA distribution of Type II and Type I fibers. N=5 in each group. **c,** The ratio of Type I fiber number o total muscle fiber. N=5 in each group. Data are presented as means ± SEM. One-way ANOVA with a post-hoc analysis **(b, upper panels, c)** was performed.

Supplementary Figure 6

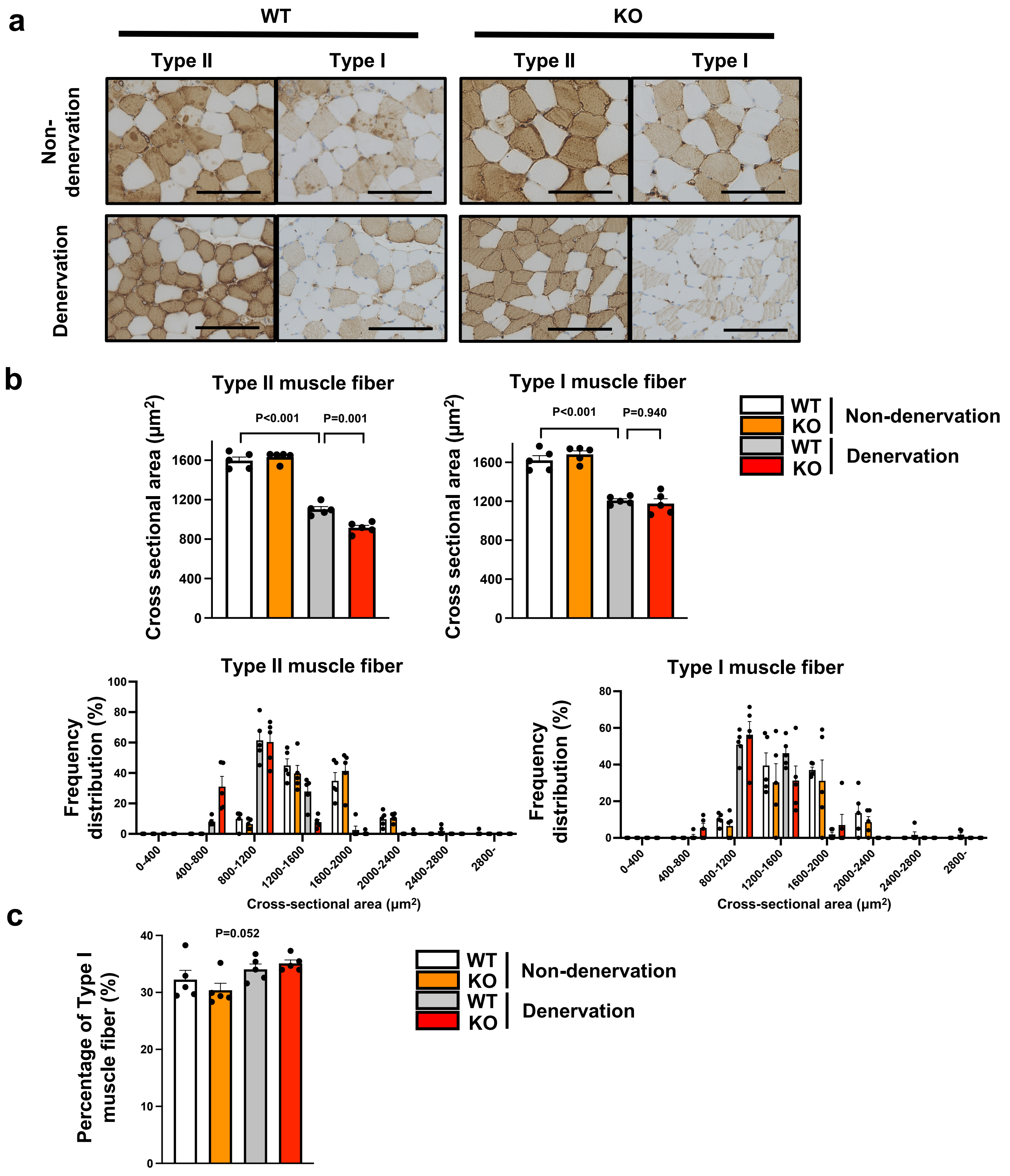

Supplementary Figure 6. Myonectin deficiency promotes denervation-induced atrophy of Type II muscle fiber in soleus muscle. Muscle fibers of Type I or Type II in denervated or non-denervated soleus muscles of WT and myonectin-KO mice, were evaluated by immune-histochemical staining. **a,** Representative immunostaining photos of Type II and Type I fibers. Scale bars show 100μm. **b,** Quantitative analysis of mean cross sectional area (CSA) and CSA distribution of Type II and Type I fibers. N=5 in each group. **c**, The ratio of Type I fiber number to total muscle fiber. N=5 in each group. Data are presented as means ± SEM. One-way ANOVA with a post-hoc analysis **(b, upper panels, c)** was performed.

Supplementary Figure 7

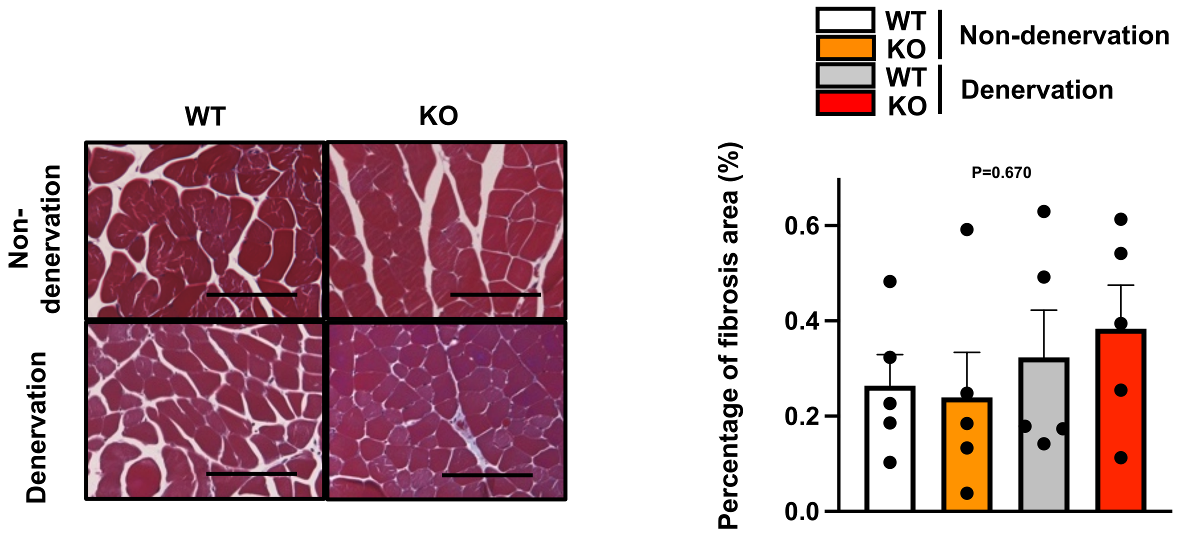

Supplementary Figure 7. Myonectin deficiency does not affect interstitial fibrosis in gastrocnemius muscles after denervation. Left panels show representative Masson trichrome staining photos in denervated or non-denervated gastrocnemius muscles of WT and myonectin-KO mice. Scale bars show 100μm. Right panel shows the quantitative analysis of fibrosis area in gastrocnemius muscle tissues. N=5 in each group. Data are presented as means ± SEM. One-way ANOVA analysis **(right panel)** was performed.

Supplementary Figure 8

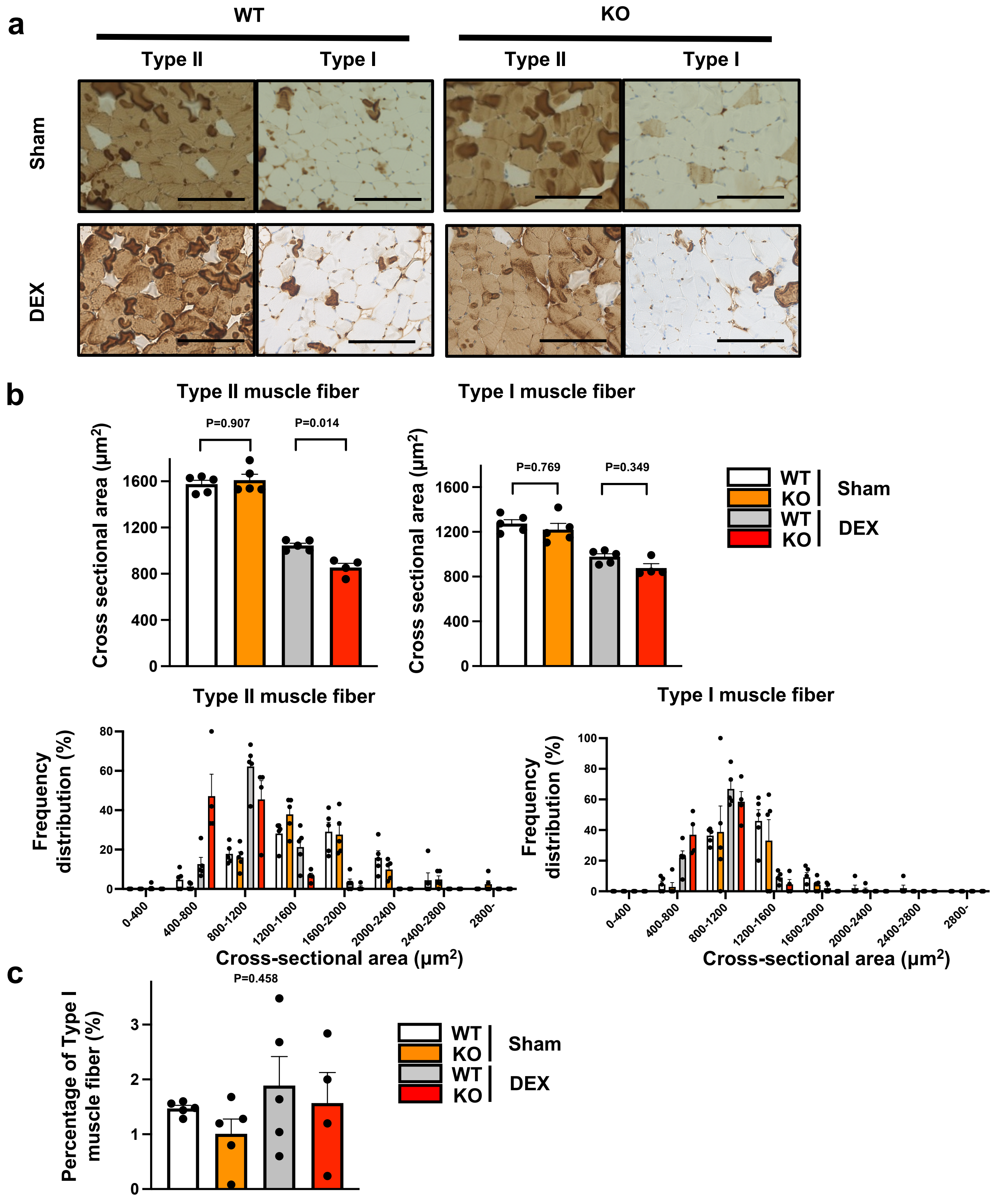

Supplementary Figure 8. Myonectin deficiency promotes Type II muscle fiber atrophy in gastrocnemius muscle after dexamethasone treatment. Muscle fibers of Type I or Type II in gastrocnemius muscles of WT and myonectin-KO mice after treatment with vehicle (sham) or dexamethasone (DEX), were evaluated by immune-histochemical staining. **a,** Representative immunostaining photos of Type II and Type I fibers. Scale bars show 100μm. **b,** Quantitative analysis of mean cross sectional area (CSA) and CSA distribution of Type II and Type I fibers. N=5 in each group. **c,** The ratio of Type I fiber number to total muscle fiber. N=5 in each group. Data are presented as means ± SEM. One-way ANOVA with a post-hoc analysis **(b, upper panels, c)** was performed.

Supplementary Figure 9

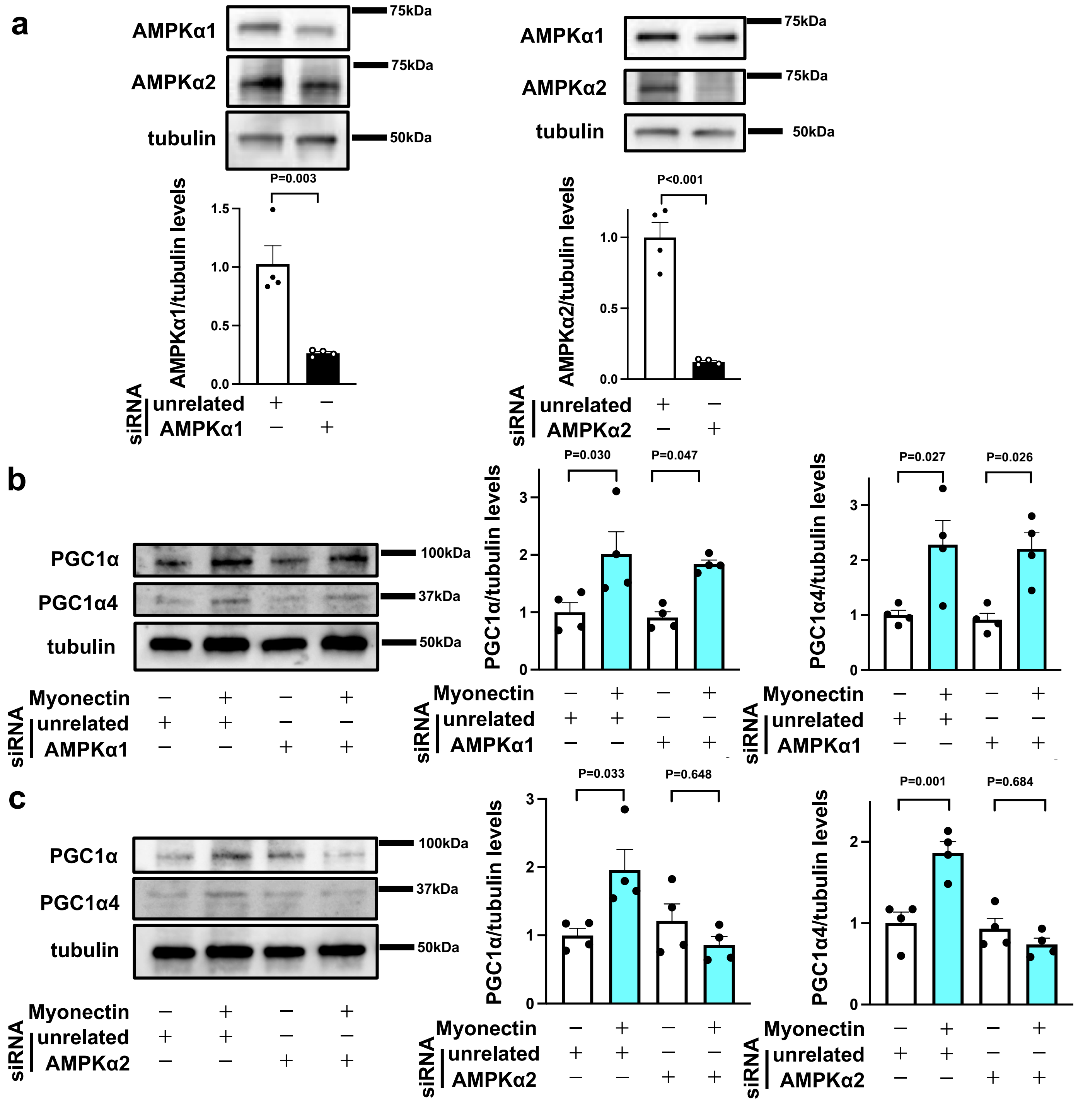

Supplementary Figure 9. Myonectin increases expression of PGC1α and PGC1α4 in C2C12 myotubes through AMPKα2-dependent pathway. a, C2C12 myotubes were treated with siRNA targeting AMPKα1 or AMPKα2, or control unrelated siRNA for 25 hours. The protein levels of AMPKα1, AMPKα2, PGC1α, PGC1α4 and tubulin were evaluated by Western blot analysis. Upper panels show representative Western blots of AMPKα1, AMPKα2 and tubulin. Lower panels show quantitative analyses of AMPKα1/tubulin and AMPKα2/tubulin signal ratios. N=4 in each group. **b,** C2C12 cells were treated with siRNA targeting AMPKα1 or unrelated siRNA for 24 hours, followed by myonectin treatment (5 μg/ml) for 1 hour. Left panels show the representative Western blots of PGC1α, PGC1α4 and tubulin. Middle and right panels show the quantitative analyses of PGC1α/tubulin and PGC1α4/tubulin signal ratios. N=4 in each group. **c,** C2C12 cells were treated with siRNA targeting AMPKα2 or unrelated siRNA for 24 hours, followed by myonectin treatment (5 μg/ml) for 1 hour. Left panels show the representative Western blots of PGC1α, PGC1α4 and tubulin. Middle and right panels show the quantitative analyses of PGC1α/tubulin and PGC1α4/tubulin signal ratios. N=4 in each group. Data are presented as means ± SEM. Two-tailed unpaired Student's t-test (**a**) and one-way ANOVA with a post-hoc analysis **(b,c)** were performed.

Supplementary Figure 10

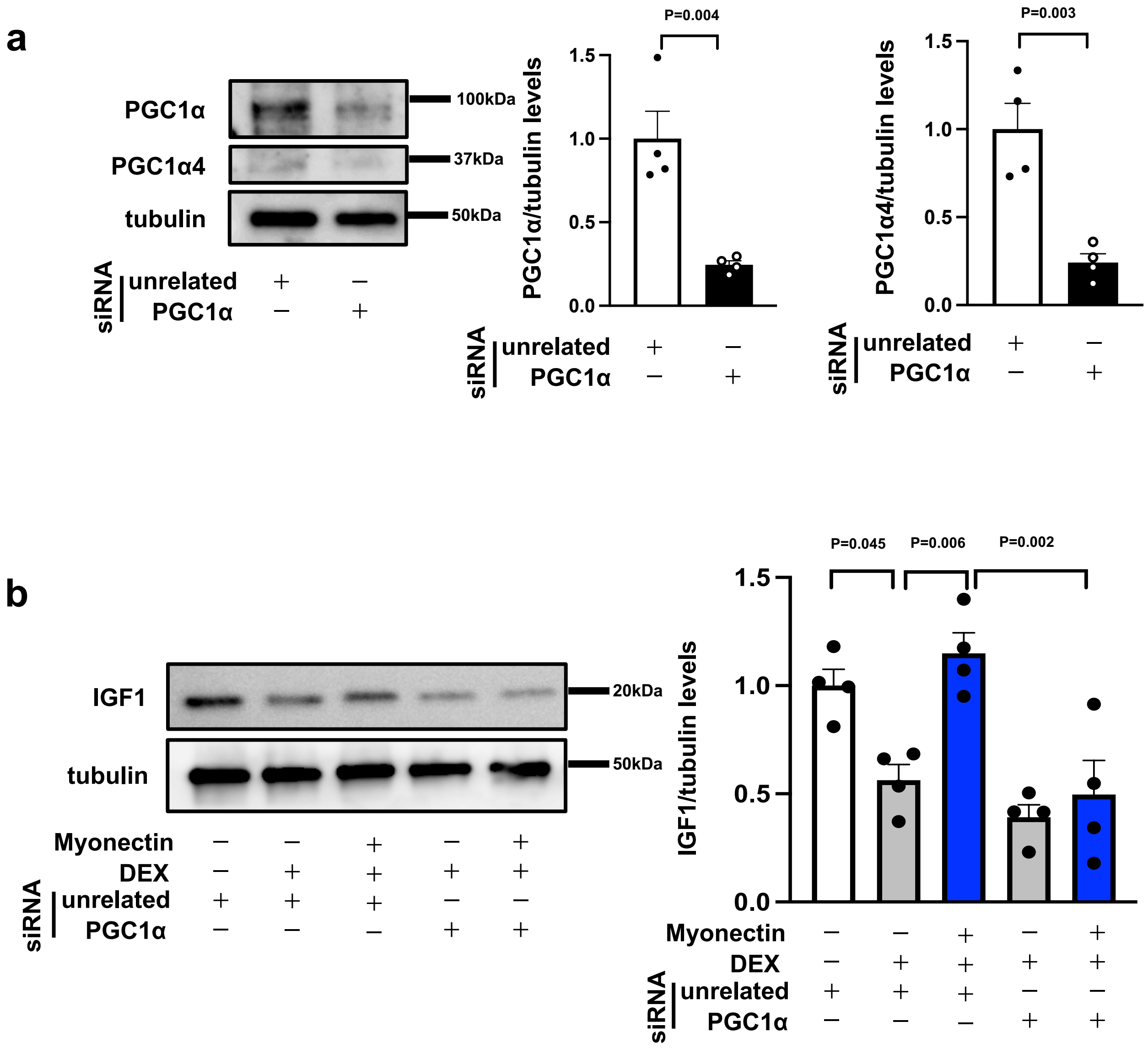

Supplementary Figure 10. Myonectin increases IGF1 expression via PGC1α-dependent pathway. a, C2C12 myotubes were treated with siRNA targeting PGC1α or control unrelated siRNA for 24 hours. The protein levels of PGC1α, PGC1α4 and tubulin were evaluated by Western blot analysis. Left panels show the representative Western blots of PGC1α, PGC1α4 and tubulin. Middle and right panels show the quantitative analyses of PGC1α/tubulin and PGC1α4/tubulin signal ratios. N=4 in each group. **b,** C2C12 myotubes were treated with siRNA targeting PGC1α or unrelated control siRNA for 24 hours and incubated in the presence or absence of myonectin protein (5 μg/ml) for 1 hour, followed by treatment with DEX (100 μM) or vehicle for 24 hours. Left panels show the representative Western blots of IGF1 and tubulin. Right panel shows the quantitative analysis of IGF1/tubulin signal ratios. N=4 in each group. Data are presented as means ± SEM. Two-tailed unpaired Student's t-test (**a**) and one-way ANOVA with a post-hoc analysis **(b)** were performed.

Supplementary Figure 11

a

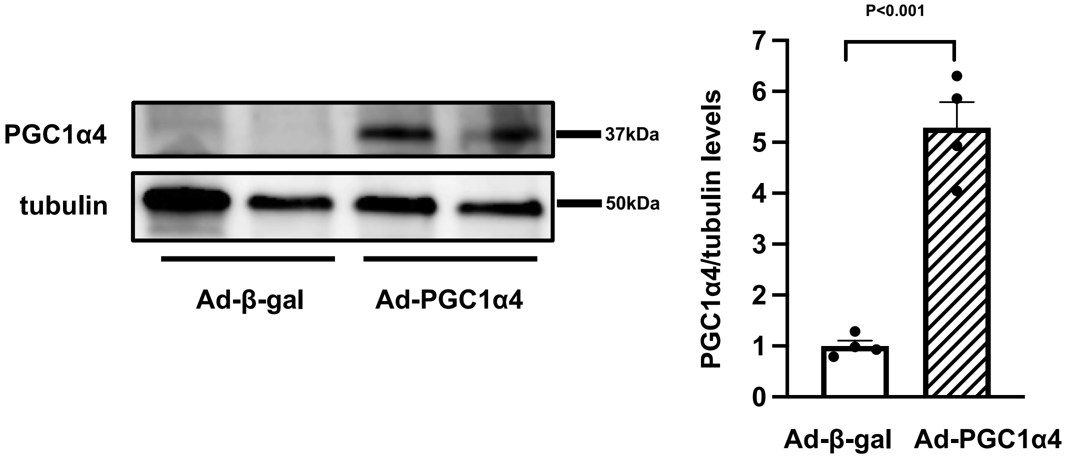

b

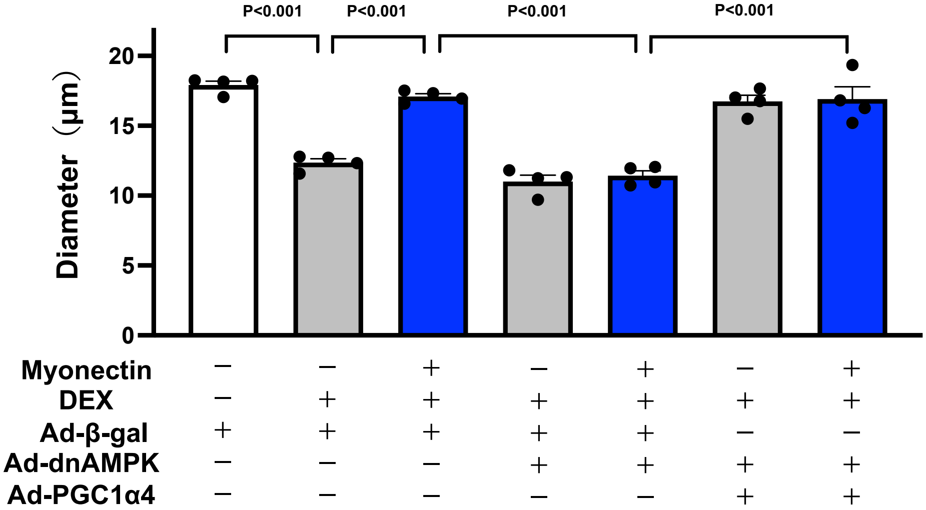

Supplementary Figure 11. PGC1α4 overexpression rescues AMPK inactivation-induced reduction of anti-atrophy effects of myonectin a, C2C12 myotubes were treated with adenoviral vectors expressing PGC1α4 (Ad-PGC1α4) or control Ad-β-gal for 49 hours. The protein levels of PGC1α4 were evaluated by Western blot analysis. Left panels show the representative Western blots of PGC1α4 and tubulin. Right panel shows the quantitative analyses of PGC1α4/tubulin signal ratios. N=4 in each group. **b,** C2C12 myotubes were pretreated with adenoviral vectors expressing dominant-negative mutant form of AMPKα2 tagged by Myc (Ad-dnAMPK), Ad-PGC1α4, or control Ad-β-gal for 24 hours and treated with myonectin protein (5 μg/ml) or vehicle for 1 hour, followed by stimulation with DEX (100 μM) or vehicle for 24 hours. Quantitative analysis of myotube diameter is shown. N=4 in each group. Data are presented as means ± SEM. Two-tailed unpaired Student's t-test (**a**) and one-way ANOVA with a post-hoc analysis **(b)** were performed.

Supplementary Figure 12

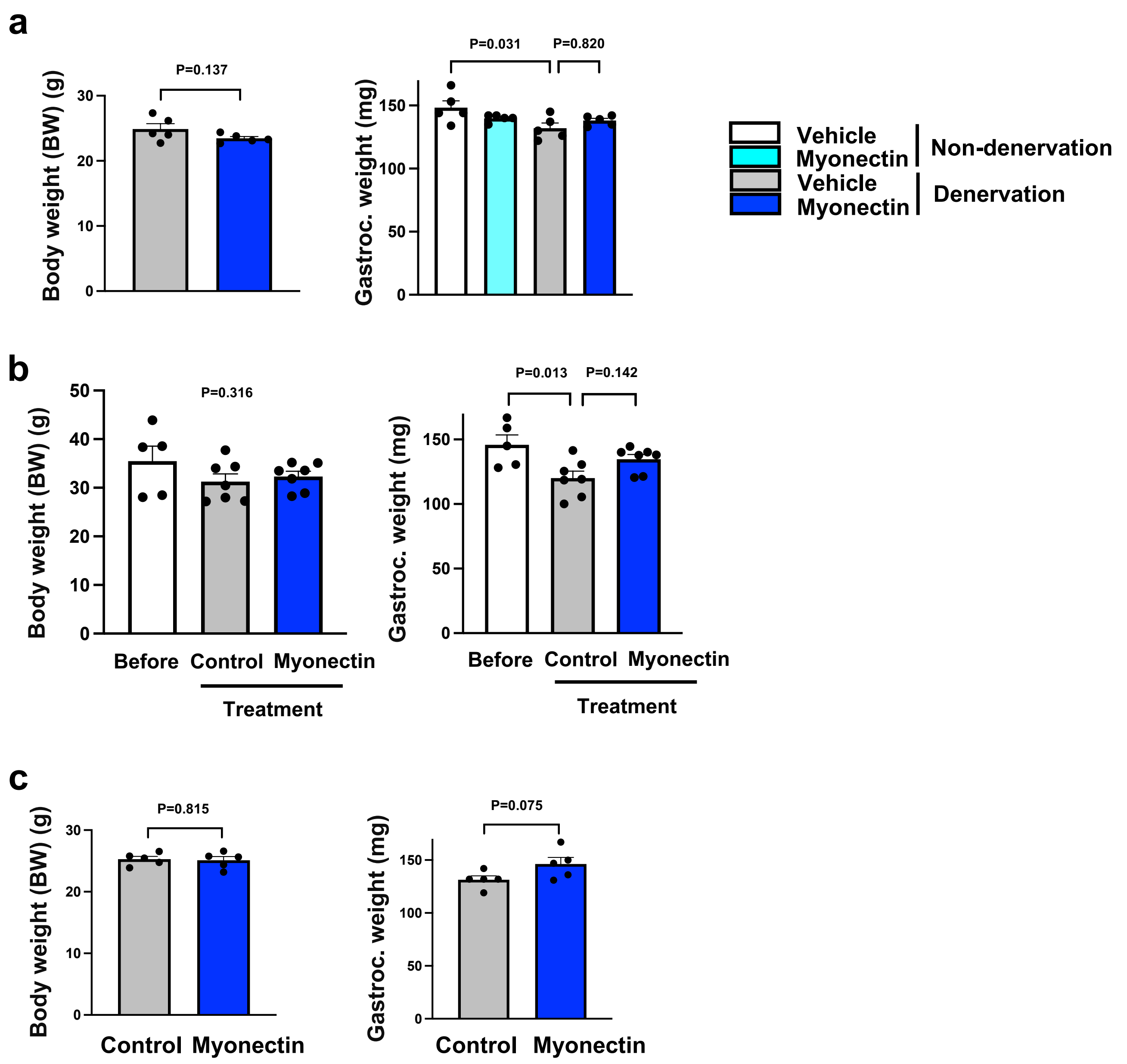

Supplementary Figure 12. Effects of myonectin on body and muscle weights in WT, SAMP8 and mdx mice. a, WT mice were subjected to sciatic denervation-induced muscle atrophy. Gelatin hydrogel impregnated with recombinant myonectin (12 μg) or vehicle was injected into the fascia of the denervated gastrocnemius muscle just before denervation. At 5 days after sciatic nerve denervation, the denervated and non-denervated gastrocnemius muscles of myonectin-treated or vehicle-treated WT mice were used for analysis. Body weights after denervation, and non-denervated or denervated gastrocnemius (Gastroc.) muscle weights are shown. N=5 in each group. **b,** Ad-myonectin (myonectin) or Ad-β-gal (control) was intra-muscularly injected into SAMP8 mice at the age of 33 weeks. At 4 weeks after treatment with myonectin or control, SAMP8 mice were sacrificed. Body weights and Gastroc. muscle weights of SAMP8 mice before treatment or after treatment with myonectin or control are shown. Before group at the age of 33 weeks: N=5, control-treated and myonectin-treated groups at the age of 37 weeks: N=7 in each group.
c, Ad-myonectin (myonectin) or Ad-β-gal (control) was injected into gastrocnemius muscles of mdx mice at the age of 4 weeks. At 4 weeks after treatment with myonectin or control, mdx mice were sacrificed. Body weights and Gastroc. muscle weights of control-treated and Ad-myonectin-treated mdx mice are shown. N=5 in each group. Data are presented as means ± SEM. Two-tailed unpaired Student's t-test (**a, left panel, c)** and one-way ANOVA with a post-hoc analysis **(a, right panel, b)** were performed.

Supplementary Figure 13

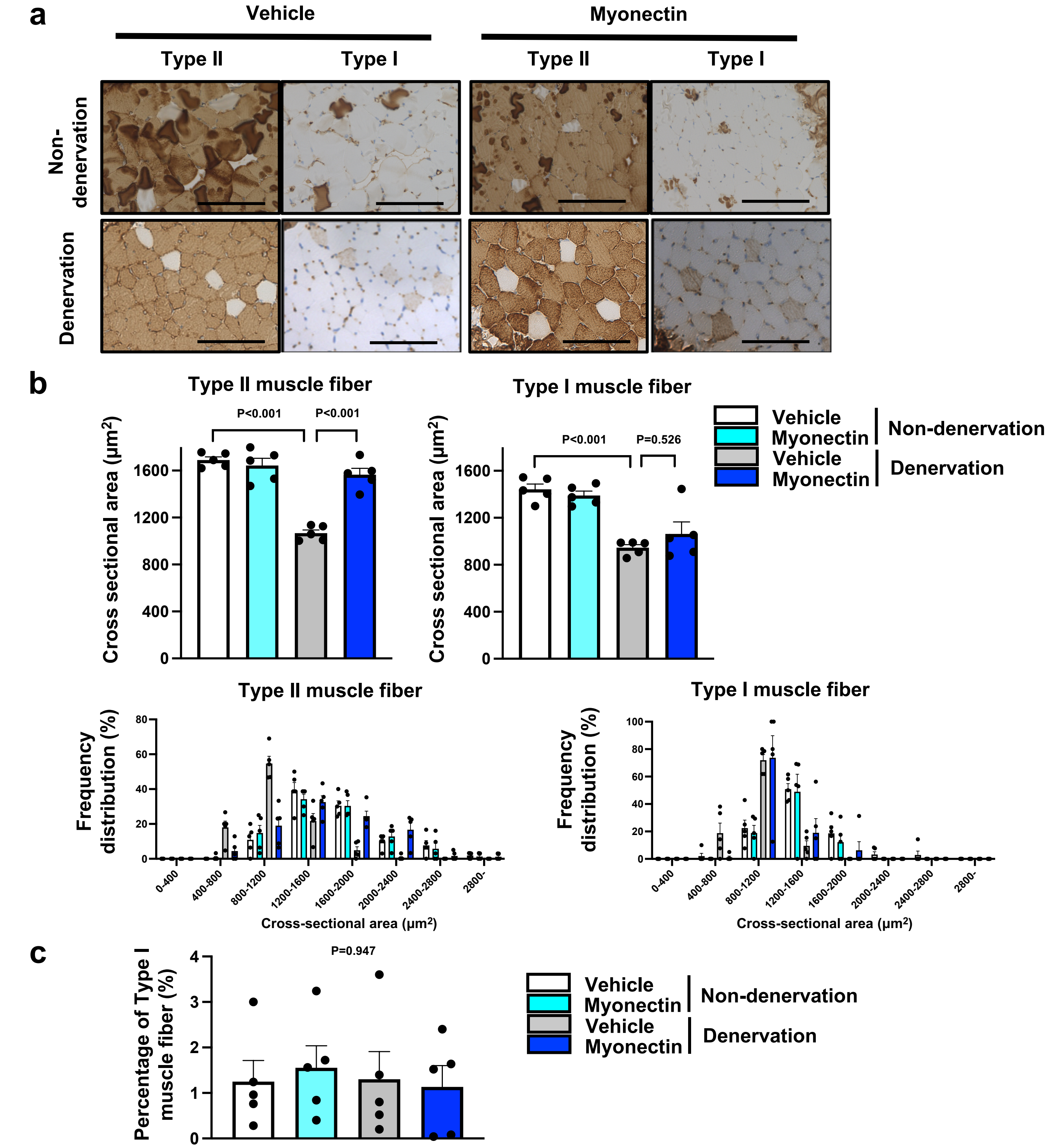

Supplementary Figure 13. Myonectin administration suppresses denervation-induced atrophy of Type II muscle fiber in gastrocnemius muscle. WT mice were subjected to sciatic denervation-induced muscle atrophy. Gelatin hydrogel impregnated with recombinant myonectin (12 μg) or vehicle was injected into the fascia of the denervated gastrocnemius muscle just before denervation. At 5 days after sciatic nerve denervation, the denervated and non-denervated gastrocnemius muscles of myonectin-treated or vehicle-treated WT mice were used for analysis. Muscle fibers of Type I or Type II in denervated or non-denervated gastrocnemius muscles of WT mice after treatment with myonectin or vehicle. **a,** Representative immunostaining photos of Type II and Type I fibers. Scale bars show 100μm. **b,** Left and right panels show the quantitative analysis of mean cross sectional area (CSA) and CSA distribution of Type II and Type I fibers. N=5 in each group. **c,** The ratio of Type I fiber number to total muscle fiber. N=5 in each group. Data are presented as means ± SEM. One-way ANOVA with a post-hoc analysis **(b, upper panels, c)** was performed.

Supplementary Figure 14

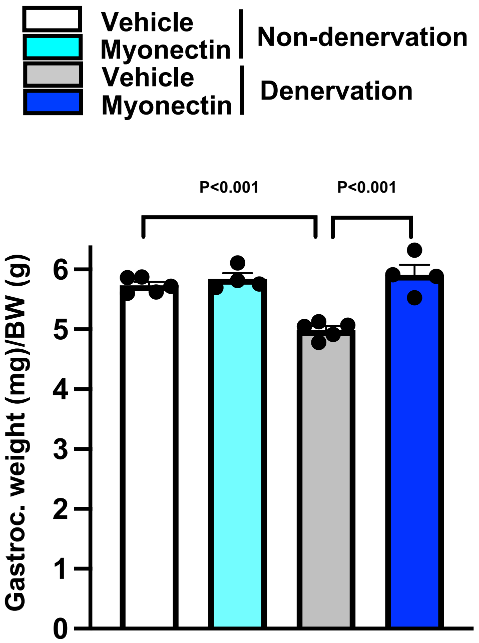

Supplementary Figure 14. Supplementation of myonectin protein one day after denervation restores denervation-induced muscle atrophy. WT mice at the age of 8-10 weeks were subjected to sciatic denervation-induced muscle atrophy operation. Gelatin hydrogel impregnated with recombinant myonectin (12 μg) or vehicle was injected into the fascia of the denervated gastrocnemius muscle at 1 day after denervation. At 5 days after sciatic nerve denervation, the denervated and non-denervated gastrocnemius muscles of myonectin-treated or vehicle-treated WT mice were used for analysis. The ratio of gastrocnemius muscle weight to body weight of myonectin-treated or vehicle-treated WT mice is shown. N=5 in each group. Data are presented as means ± SEM. One-way ANOVA with a post-hoc analysis was performed.

Supplementary Figure 15

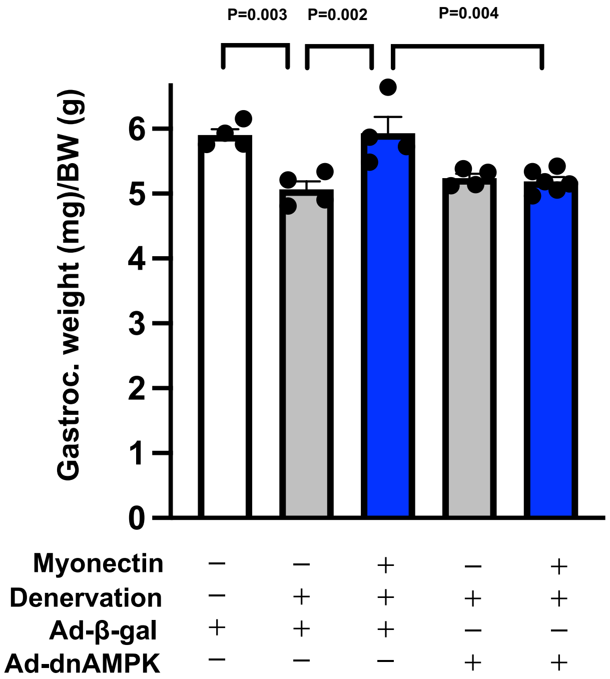

Supplementary Figure 15. Effect of myonectin on muscle weights of WT mice subjected to nerve denervation after treatment with Ad-dnAMPK or Ad-β-gal. Ad-dnAMPK or Ad-β-gal was intramuscularly administered to the gastrocnemius muscle of WT mice at 3 days before setting of gelatin hydrogel impregnated with recombinant myonectin (12 μg) or vehicle. Gastrocnemius (Gastroc.) muscle weight/body weight (BW) ratio was evaluated. N=5 in each group. Data are presented as means ± SEM. One-way ANOVA with a post-hoc analysis was performed.

Supplementary Figure 16

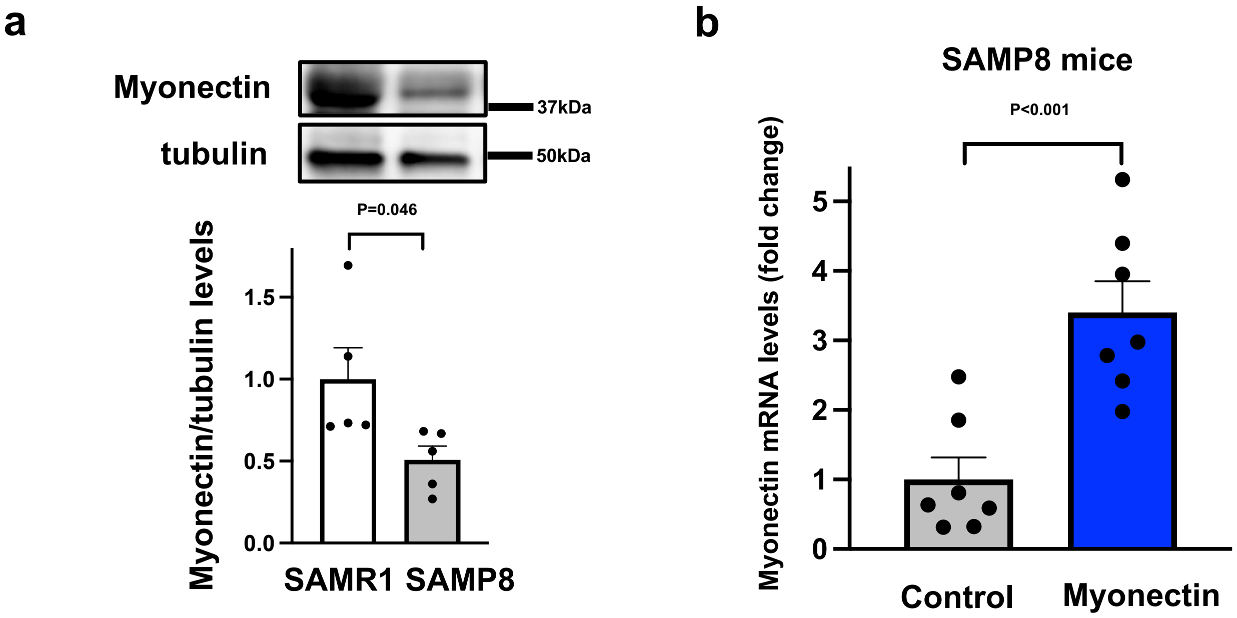

Supplementary Figure 16. SAMP8 mice show reduced protein levels of myonectin in gastrocnemius muscle. a, The protein levels of myonectin and tubulin were evaluated by Western blot analysis. Upper panels show the representative Western blots of myonectin and tubulin in gastrocnemius muscles of SAMP8 and control SAMR1 mice at the age of 37 weeks. Lower panels show quantitative analysis of myonectin/tubulin signal ratios in gastrocnemius muscles of SAMP8 and control SAMR1 mice. N=5 in each group. **b,** Ad-myonectin (myonectin) or Ad-β-gal (control) was intra-muscularly injected into SAMP8 mice at the age of 33 weeks. At 4 weeks after treatment with myonectin or control, SAMP8 mice were sacrificed. The mRNA levels of myonectin were evaluated by quantitative real time PCR methods. N=7 in each group. Data are presented as means ± SEM. Two-tailed unpaired Student's t-test **(a,b)** was performed.

Supplementary Figure 17

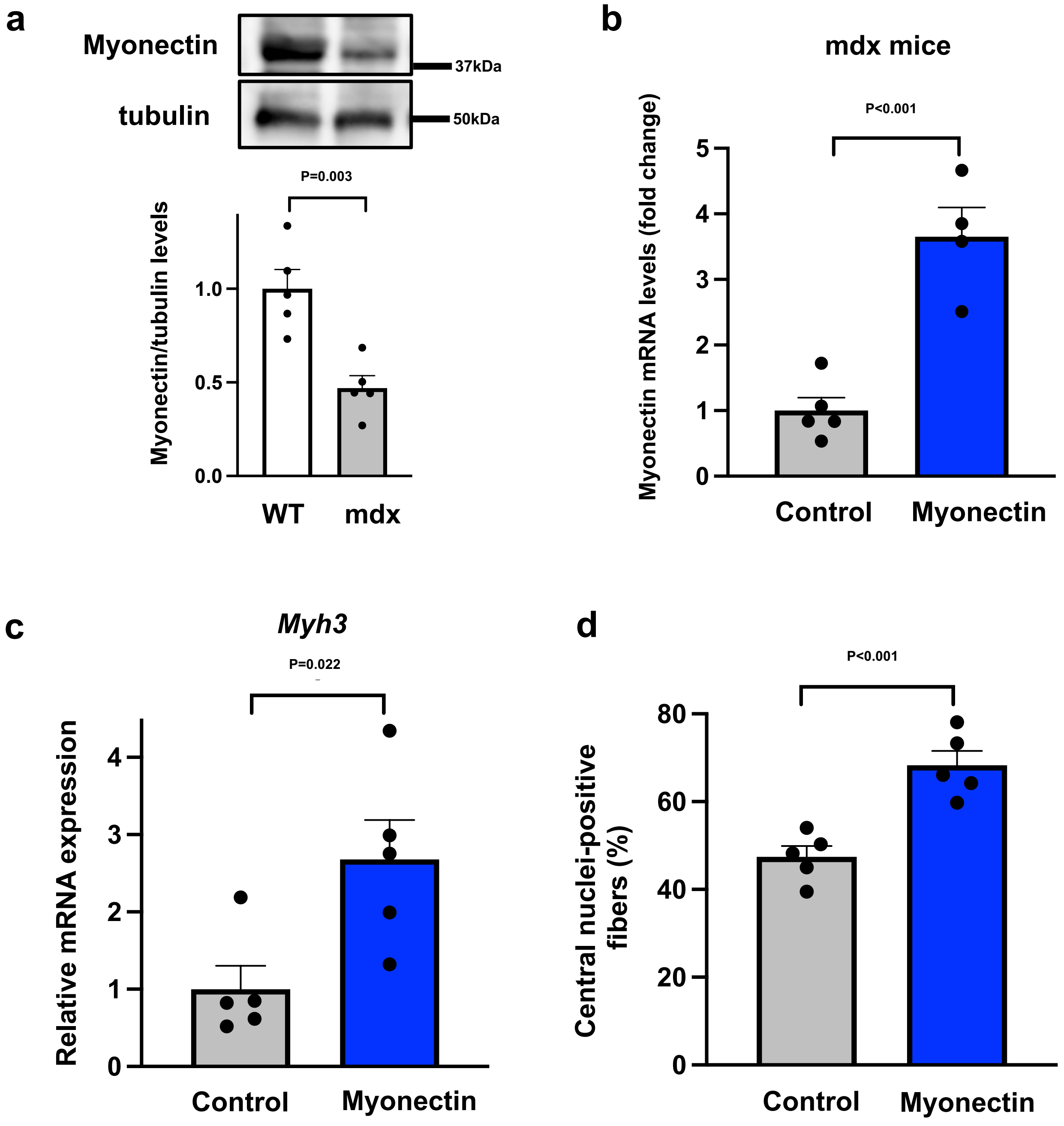

Supplementary Figure 17. Mdx mice show reduced protein levels of myonectin in gastrocnemius muscle. a, The protein levels of myonectin and tubulin were evaluated by Western blot analysis. Left panels show the representative Western blots and quantitative analysis of myonectin/tubulin signal ratios in gastrocnemius muscles of mdx and wild-type (WT) mice at the age of 8 weeks. N=5 in each group. **b, c and d,** Ad-myonectin (myonectin) or Ad-β-gal (control) was injected into gastrocnemius muscles of mdx mice at the age of 4 weeks. At 4 weeks after treatment with myonectin or control, mdx mice were sacrificed, and the gastrocnemius muscles were used for analysis. **b,** The expression levels of myonectin were evaluated by quantitative real time PCR methods. N=5 in each group. **c,** The mRNA level of myosin heavy chain embryonic (*Myh3*) is evaluated by quantitative PCR analysis. N=5 in each group. **d,** The percentage of fibers with central nuclei-positive cells divided by total cells is shown. N=5 in each group. Data are presented as means ± SEM. Two-tailed unpaired Student's t-test **(a,b,c,d)** was performed.

Supplementary Table

Primers used for quantitative RT-PCR

Fam132b:	forward	5'- TTATCCCATCTGAGGTTCTG -3'
	reverse	5'- CAGATGGCTCTCTCGCT -3'
Pgc1α:	forward	5’- TGATGTGAATGACTTGGATACAGACA -3'
	reverse	5’- GCTCATTGTTGTACTGGTTGGATATG -3'
Pgc1α4:	forward	5’- TCACACCAAACCCACAGAAA -3'
	reverse	5’- CTGGAAGATATGGCACAT -3'
Fbxo32:	forward	5'- GAGGCAGATTCGCAAGCGTTTGAT -3'
	reverse	5'- TCCAGGAGAGAATGTGGCAGTGTT -3'
Trim63:	forward	5'- AGTGTCCATGTCTGGAGGTCGTTT -3'
	reverse	5'- ACTGGAGCACTCCTGCTTGTAGAT -3'
Mstn:	forward	5'- AAGTCTCTCTCCGGGACCTCTT -3'
	reverse	5'- TGTAACCTTCCCAGGACCAG -3'
Myod1:	forward	5'- CTTCTATCGCCGCCACTC -3'
	reverse	5'- AAGTCGTCTGCTGTCTCAA -3'
Myog:	forward	5'- CCAACCCAGGAGATCATTTG -3'
	reverse	5'- ACGATGGACGTAAGGGAGTG -3'
Tfam:	forward	5'- CCAAGTCAGCTGATGGGTATGG -3'
	reverse	5'- CCTGAGCCGAATCATCCTTTGC -3'
Sirt1:	forward	5'- TCCTCACTAATGGCTTTCATTCCTG -3'
	reverse	5'- GTGCCAATCATGAGATGTTGCTG -3'
Nrf1:	forward	5'- GAGCACGGAGTGACCCAAAC -3'
	reverse	5'- TGTACGTGGCTACATGGACCT -3'
Nfe2l2:	forward	5'- TTGGCAGAGACATTCCCATTTGTA -3'

	reverse	5'- AGTCATGGCTGCCTCCAGAGA -3'
Myh3:	forward	5'- ACGACAACTCGTCTCG -3'
	reverse	5'- TTGGTCGTAATCAGCA -3'
36B4:	forward	5'- GCTCCAAGCAGATGCAGCA -3'
	reverse	5'- CCGGATGTGAGGCAGCAG -3'

Fam132b: myonectin, *Pgc1α*: PGC1α, *Mstn*: myostatin, *Nfe2l2*: Nuclear factor erythroid 2-related factor 2, *Myh3*: myosin heavy chain embryonic.
