# Supplementary material for: Myonectin protects against skeletal muscle dysfunction in male mice through activation of AMPK/PGC1α pathway
Source: Nat Commun. 2023 Aug 4;14:4675. doi: 10.1038/s41467-023-40435-2 (PMC10403505; doi:10.1038/s41467-023-40435-2)
Supplement: Supplementary file 3 — Reporting Summary [file 41467_2023_40435_MOESM3_ESM.pdf]

nature portfolio

Corresponding author(s): Koji Ohashi and Noriyuki Ouchi

Last updated by author(s): 2023/07/18

Reporting Summary

Nature Portfolio wishes to improve the reproducibility of the work that we publish. This form provides structure for consistency and transparency in reporting. For further information on Nature Portfolio policies, see our Editorial Policies and the Editorial Policy Checklist.

Statistics

For all statistical analyses, confirm that the following items are present in the figure legend, table legend, main text, or Methods section.

n/a	Confirmed	
☐	☒	The exact sample size (*n*) for each experimental group/condition, given as a discrete number and unit of measurement
☐	☒	A statement on whether measurements were taken from distinct samples or whether the same sample was measured repeatedly
☐	☒	The statistical test(s) used AND whether they are one- or two-sided<br>*Only common tests should be described solely by name; describe more complex techniques in the Methods section.*
☐	☒	A description of all covariates tested
☐	☒	A description of any assumptions or corrections, such as tests of normality and adjustment for multiple comparisons
☐	☒	A full description of the statistical parameters including central tendency (e.g. means) or other basic estimates (e.g. regression coefficient) AND variation (e.g. standard deviation) or associated estimates of uncertainty (e.g. confidence intervals)
☐	☒	For null hypothesis testing, the test statistic (e.g. *F*, *t*, *r*) with confidence intervals, effect sizes, degrees of freedom and *P* value noted<br>*Give P values as exact values whenever suitable.*
☒	☐	For Bayesian analysis, information on the choice of priors and Markov chain Monte Carlo settings
☒	☐	For hierarchical and complex designs, identification of the appropriate level for tests and full reporting of outcomes
☒	☐	Estimates of effect sizes (e.g. Cohen's *d*, Pearson's *r*), indicating how they were calculated

Our web collection on statistics for biologists contains articles on many of the points above.

Software and code

Policy information about availability of computer code

Data collection	Illumina NovaSeq 6000. BioRad real-time PCR detection system (TOYOBO). Small-animal grip strength meter (Columbus Co., Largo, FL). Wheel running system (Melquest Ltd., Toyama).
Data analysis	IBM SPSS Statistics 28 software (SPSS Inc). Image J software ver1.53k (National Institute of Health). FastQC (Version: 0.10.0). TrimGalore! (0.6.4). STAR (2.7.3a). HTSeq-count(version 0.11.2). DESeq2 package (1.32.0).<br>R (4.1.0). Database for Annotation, Visualization and Integrated Discovery (DAVID, v6.8)

For manuscripts utilizing custom algorithms or software that are central to the research but not yet described in published literature, software must be made available to editors and reviewers. We strongly encourage code deposition in a community repository (e.g. GitHub). See the Nature Portfolio guidelines for submitting code & software for further information.

Data

Policy information about availability of data

All manuscripts must include a data availability statement. This statement should provide the following information, where applicable:

- Accession codes, unique identifiers, or web links for publicly available datasets
- A description of any restrictions on data availability
- For clinical datasets or third party data, please ensure that the statement adheres to our policy

All the data are included in the main text, Supplementary information and Source data. Any additional information of data can be available from the corresponding

authors on request.
The RNA sequencing data generated in this study have been deposited in the Gene Expression Omnibus (GEO) database under accession code GSE233328 [https://www.ncbi.nlm.nih.gov/geo/query/acc.cgi?acc=GSE233328].
Source data are provided with this paper.

Research involving human participants, their data, or biological material

Policy information about studies with human participants or human data. See also policy information about sex, gender (identity/presentation), and sexual orientation and race, ethnicity and racism.

Reporting on sex and gender	N/A.
Reporting on race, ethnicity, or other socially relevant groupings	N/A.
Population characteristics	N/A.
Recruitment	N/A.
Ethics oversight	N/A.

Note that full information on the approval of the study protocol must also be provided in the manuscript.

Field-specific reporting

Please select the one below that is the best fit for your research. If you are not sure, read the appropriate sections before making your selection.

☒ Life sciences ☐ Behavioural & social sciences ☐ Ecological, evolutionary & environmental sciences

For a reference copy of the document with all sections, see nature.com/documents/nr-reporting-summary-flat.pdf

Life sciences study design

All studies must disclose on these points even when the disclosure is negative.

Sample size	Sample size was determined in accordance to standard practices in this field of research and based on previous analyses and experience with similar experimental paradigms (PMID: 35501350, PMID: 33658508).
Data exclusions	No data were excluded.
Replication	All studies were not replicated but they included sufficient numbers to account for biological variability. In addition, multiple techniques and models were used to validate the same findings, each with 4 or more biological replicated.
Randomization	Randomization was not a relevant feature as a uniform set of animal or cultured cells were applied to all experiments.
Blinding	Blinding was not a relevant feature as a uniform set of animal or cultured cells were applied to all experiments.

Reporting for specific materials, systems and methods

We require information from authors about some types of materials, experimental systems and methods used in many studies. Here, indicate whether each material, system or method listed is relevant to your study. If you are not sure if a list item applies to your research, read the appropriate section before selecting a response.

Materials & experimental systems

n/a	Involved in the study	
☐	☒	Antibodies
☐	☒	Eukaryotic cell lines
☒	☐	Palaeontology and archaeology
☐	☒	Animals and other organisms
☒	☐	Clinical data
☒	☐	Dual use research of concern
☒	☐	Plants

Methods

n/a	Involved in the study	
☒	☐	ChIP-seq
☒	☐	Flow cytometry
☒	☐	MRI-based neuroimaging

Antibodies

Antibodies used	Antibodies against phosphorylated AMPK (Thr172)(Cat. 2531S)(diluted at 1:1000), AMPK (Cat. 2532S)(diluted at 1:1000), phosphorylated ACC (Ser79)(Cat. 3661)(diluted at 1:1000), ACC (Cat. 3662)(diluted at 1:1000), COX4 (Cat. 4844)(diluted at 1:1000) and α-tubulin (Cat. 2144S)(diluted at 1:1000) were purchased from Cell Signaling Technology. Antibodies against AMPKα1 (Cat. ab3759)(diluted at 1:1000), AMPKα2 (Cat. ab3760)(diluted at 1:1000) and total OXPHOS (Cat. ab110413)(diluted at 1:500) were purchased from Abcam. Antibody against myonectin was purchased from Santa Cruz Biotechnology (Cat. sc-246565)(diluted at 1:2500). Antibody against IGF1 (Cat. AF-791) was purchased from R&D Systems, Inc.(diluted at 1:500). Antibody against PGC1α (PGC1α and PGC1α4) was purchased from Calbiochem (Cat.ST1202-1SETCN). Mouse monoclonal anti-slow myosin (Clone NOQ7.5.4D) (Cat. SAB4200670)(diluted at 1:10000) and mouse monoclonal anti-fast myosin (Clone MY-32)(Cat. M4276)(diluted at 1:800) were purchased from Sigma. Goat anti-rabbit IgG HRP-linked antibody (Cat. 7074) and horse anti-mouse IgG HRP-linked antibody (Cat. 7076) were purchased from Cell Signaling Technology?(diluted at 1:5000). Bovine anti-goat IgG HRP-linked antibody (Cat. sc-2384) was purchased from Santa Cruz (diluted at 1:2000).
Validation	All antibodies in this study were used and validated according to the provided data sheets and reference for the specific technique (Western blotting, immunostaining) fond directly on the manufacture's website.<br>1. phosphorylated AMPK (Thr172): https://www.cellsignal.jp/products/primary-antibodies/phospho-ampka-thr172-antibody/2531<br>2. AMPK: https://www.cellsignal.jp/products/primary-antibodies/ampka-antibody/2532<br>3. phosphorylated ACC (Ser79): https://www.cellsignal.jp/products/primary-antibodies/phospho-acetyl-coa-carboxylase-ser79-antibody/3661<br>4. ACC: https://www.cellsignal.jp/products/primary-antibodies/acetyl-coa-carboxylase-antibody/3662<br>5. COX4: https://www.cellsignal.com/products/primary-antibodies/cox-iv-antibody/4844<br>6. α-tubulin: https://www.cellsignal.jp/products/primary-antibodies/a-tubulin-antibody/2144<br>7. AMPKα1: https://www.abcam.co.jp/products/primary-antibodies/ampk-alpha-1-antibody-ab3759.html<br>8. AMPKα2: https://www.abcam.co.jp/products/primary-antibodies/ampk-alpha-2-antibody-ab3760.html<br>9. OXPHOS: https://www.abcam.co.jp/products/panels/total-oxphos-rodent-wb-antibody-cocktail-ab110413.html<br>10. Myonectin: https://datasheets.scbt.com/sc-246565.pdf<br>11. IGF-1: https://www.rndsystems.com/products/mouse-igf-i-igf-1-antibody_af791<br>12. PGC1α (PGC1α and PGC1α4): https://www.merckmillipore.com/JP/ja/product/Anti-PGC-1-Mouse-mAb-4C1.3,EMD_BIO-ST1202#anchor_PDS<br>13. anti-slow myosin (Clone NOQ7.5.4D): https://www.sigmaaldrich.com/specification-sheets/389/233/SAB4200670-BULK___SIGMA____.pdf<br>14. anti-fast myosin (Clone MY-32): https://www.sigmaaldrich.com/specification-sheets/165/088/M4276-BULK_________SIGMA____.pdf<br>15. Goat anti-rabbit IgG HRP-linked antibody: https://www.cellsignal.jp/products/secondary-antibodies/anti-rabbit-igg-hrp-linked-antibody/7074<br>16. horse anti-mouse IgG HRP-linked antibody: https://www.cellsignal.com/products/secondary-antibodies/anti-mouse-igg-hrp-linked-antibody/7076<br>17. Bovine anti-goat IgG HRP-linked antibody: https://datasheets.scbt.com/sc-2384.pdf

Eukaryotic cell lines

Policy information about cell lines and Sex and Gender in Research

Cell line source(s)	C2C12 cells were purchased from American Type Culture Collection (ATCC) (CRL-1772).
Authentication	C2C12 cells were purchased from authenticated vendors, and morphology was also confirmed visually prior to use.
Mycoplasma contamination	Mycoplasma free tested by vendors.
Commonly misidentified lines (See ICLAC register)	No commonly misidentified cell lines were used in this study.

Animals and other research organisms

Policy information about studies involving animals; ARRIVE guidelines recommended for reporting animal research, and Sex and Gender in Research

Laboratory animals	Male C57BL/6J (The Jackson Laboratory, JAX#000664) mice were utilized at 8-10 weeks or 80 weeks of age.<br>Male myonectin knockout mice (Lexicon Pharmaceuticals) in a background of C57BL/6 were utilized at 8-10 weeks or 80 weeks of age.<br>Male human skeletal a-actin promoter (HSA) promoter-driven dominant negative mutant form of AMPK transgenic (DN-AMPK Tg) mice in a background of C57BL/6 were purchased from JCRB (Japanese Collection of Research Bioresources Cell Bank) Laboratory Animal Resource Bank at NIBIOHN (National Institute of Biomedical Innovation, Health and Nutrition. Osaka, Japan). This strain mice were utilized at 8-10 weeks of age.<br>Male mdx mice in a background of C57BL/6 were purchased from Chubu Kagaku Shizai Co.,Ltd.(Nagoya, Japan), and utilized at 4 weeks of age.<br>Male SAMP8 mice (SAMP8/TaSlc) and control SAMR1/TaSlc in a background of AKR/J mice were purchased from Japan SLC, Inc. (Hamamatsu, Japan), and utilized at 33 weeks of age.<br>All mice were housed at 20-22° and 50% relative humidity in a 12 h light/dark cycle. Mice had free access to water and standard chow (CE-2, CLEA Japan Inc.).

Wild animals	No wild animals were used in this study.
Reporting on sex	Male mice were used for all experiments, because the previous our and other group's studies were performed using male mice and be easy to confirm the relevance of study results. Thus, future study will be required to confirm whether our findings are applied to both sex using female mice.
Field-collected samples	No field-collected samples were used in this study.
Ethics oversight	All animal procedures were approved by the Institutional Animal Care and Use Committee of Nagoya University School of Medicine.

Note that full information on the approval of the study protocol must also be provided in the manuscript.
